# Donor‐induced conformational gating and substrate‐assisted catalysis in *α* ‐1,3‐galactosyltransferase

**DOI:** 10.1002/pro.70770

**Published:** 2026-08-15

**Authors:** Javier A. Linares‐Pastén, Antoni Planas

**Affiliations:** ^1^ Biotechnology and Applied Microbiology, Department of Process and Life Sciences Engineering LTH, Lund University Lund Sweden; ^2^ Laboratory of Biochemistry, Institut Químic de Sarrià University Ramon Llull Barcelona Spain; ^3^ Chemistry section Royal Academy of Sciences and Arts of Barcelona Barcelona Spain

**Keywords:** Galili epitope, retaining glycosyltransferases, UDP‐galactose, *α*‐1,3‐galactosyltransferase

## Abstract

Retaining glycosyltransferases catalyze the formation of stereochemically conserved glycosidic bonds through mechanisms that remain debated. Using bovine *α*1,3‐galactosyltransferase (*α*3GalT) as a model, we combine mutagenesis, equilibrium unfolding, kinetics, and molecular dynamics simulations to understand how donor‐induced loop ordering promotes catalysis. Alanine‐scanning mutagenesis of the C‐terminal loop (Thr358‐Val368) identified Lys359, Tyr361, and Arg365 as critical for donor binding, catalysis, and ligand‐dependent stabilization. In addition, D225A and E317A were inactive and showed minimal ligand‐induced stabilization, consistent with impaired metal binding and substrate stabilization, respectively. Donor binding induces an ordered conformation in the C‐terminus, reducing its local flexibility by 30% and pre‐organizing the active site for catalysis. MD‐derived energy profiles differed markedly for the donor (UDP‐Gal) and acceptor (lactose) in the ternary complex. In this context, experimental apparent *K*ₘ values indicate higher donor affinity than acceptor affinity. Our results show that donor binding stabilizes the C‐terminal loop, assembling a competent complex for catalysis. These findings support a general coupling between conformational gating, donor stabilization, and the catalytic mechanism in retaining GT‐A‐fold enzymes.

## 1 INTRODUCTION

Glycosyltransferases (GTs) catalyze the formation of glycosidic bonds (EC 2.4.x.y) in a wide variety of glycoconjugates. In particular, glycolipids and glycoproteins on the eukaryotic cell surface play important physiological roles in mediating interactions with pathogens, such as bacteria and viruses, as well as cellular recognition, signaling, and interactions with molecules like hormones and toxins. The *α*‐1,3‐glycosyltransferase (*α*3GalT) (EC 2.4.1.151) is responsible for the synthesis of the xenoantigen Gal*α*3Gal*β*4GlcNAc in many mammals, except apes, Old World monkeys, and humans (Galili et al., 1988). The terminal disaccharide Gal*α*3Gal*β*4, called Galili epitope, triggers the hyperacute (vascular) rejection (HAR) in the xenotransplantation of grafts from animals to humans due to the naturally occurring human antibodies against this epitope (Galili, 2001; Joziasse & Oriol, 1999).

Structurally, the GTs comprise four general folds: GT‐A, GT‐B, GT‐C, and the Lysozyme‐Type fold (Lairson et al., 2008; Rini et al., 2022; Taujale et al., 2021). The GT‐A consists of a single domain with a Rossmann‐like *β*/*α*/*β* architecture, typical of nucleotide‐binding proteins. Most GT‐A enzymes possess a DXD motif in which the carboxylates coordinate a divalent cation, which in turn binds to the phosphate of the donor substrate. The GT‐B architecture consists of two *β*/*α*/*β* Rossmann‐like domains, N‐ and C‐terminus, linked by a flexible linker creating an interdomain substrate‐binding cleft. GT‐B enzymes lack the DXD metal‐binding motif. The GT‐C fold occurs mainly in integral membrane glycosyltransferases and features substantial topological and mechanistic differences. It is distinguished by 8–13 transmembrane helices, placing the active site in long loop regions; most GT‐C fold enzymes use lipid‐linked sugar donors (Albuquerque‐Wendt et al., 2019; Alexander & Locher, 2023). The lysozyme‐type fold is present in some glycosyltransferases with a similar structure to that of lysozyme (Taujale et al., 2021). These enzymes also use lipid‐linked sugar donors. Recent studies have identified structures with novel folds, for example, the five‐bladed *β*‐propeller fold in mannosyltransferases of family GT108 (Sernee et al., 2019). This unique fold occurs in select membrane‐associated glycosyltransferases.

The *α*3GalT belongs to the GT‐A family and represents the “prototype” of this family. It has been structurally studied in multiple states, as evidenced by high‐resolution crystal structures available in the Protein Data Bank (PDB). These include substrate‐free forms, exemplified by PDB ID 1FG5 (Gastinel et al., 2001), and substrate‐ or ligand‐bound forms, such as 1GWW and 1GX0, which reveal interactions with donor substrates, such as UDP‐galactose, and acceptors, such as lactose and N‐acetyllactosamine (Boix et al., 2001; Boix et al., 2002). Structures of mutant forms (Jamaluddin et al., 2007), with substrate analogues (Jamaluddin et al., 2009), and ternary complexes in catalytically productive conformations provide insight into the enzyme's catalytic mechanism (Albesa‐Jové et al., 2017), emphasizing ordered substrate binding and the conformational changes essential for galactose transfer. Key conserved residues, notably the catalytic residue Glu317, have been identified through these structural studies. On the other hand, the C‐terminus region (Thr358 to Val368) involved in the active site is disordered (form I) in some of them (Gastinel et al., 2001) and is a short helix in others (form II), suggesting conformational changes associated with catalysis (Boix et al., 2001; Boix et al., 2002). Thus, the ensemble of bovine *α*3GalT structures reported, spanning from 2000 to recent years, has contributed to understanding the relationship between structure and function.

**FIGURE 1:**
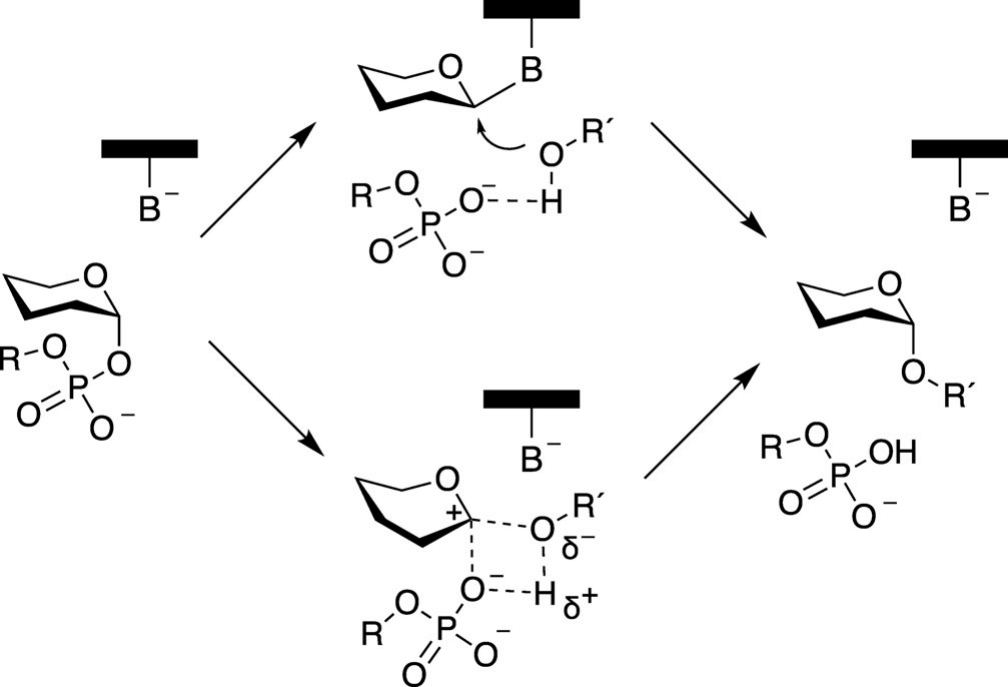
Proposed catalytic mechanisms for retaining GTs. A double displacement mechanism (top) and a front‐face mechanism (bottom).

Retaining Leloir glycosyltransferases (GTs) catalyze the transfer of sugars from nucleotide donors to diverse acceptors while conserving the donor anomeric configuration. Despite decades of work, the mechanism by which retention is achieved has remained debated, with two main proposals: a Koshland‐type double‐displacement via a covalent glycosyl–enzyme intermediate, and a front‐side “internal return” (SNi‐like) substitution in which leaving‐group departure and nucleophilic attack occur from the same face of the sugar (Figure 1) (Ardevol et al., 2016; Tvaroška, 2015). Initial chemical rescue studies on the E317A mutant of *α*3GalT provided compelling evidence for a double‐displacement mechanism, identifying Glu317 as a likely catalytic nucleophile and involving a covalent intermediate, while still leaving open the possibility of an SNi mechanism (Monegal & Planas, 2006). The challenge is exemplified by recent structural and computational studies that have begun to capture catalytically competent Michaelis complexes and to delineate transition‐state features for multiple GT families (Gómez et al., 2013).

Multiple lines of evidence now support SNi‐type reactivity in several retaining GTs. In the GT8 enzyme LgtC, hybrid QM/MM simulations showed transfer via an oxocarbenium‐ion‐like transition state with front‐side approach and further implicated the donor's *β*‐phosphate as a general base for acceptor activation (Gómez et al., 2015). Experimentally, a native ternary complex of the GT‐A enzyme glucosyl‐3‐phosphoglycerate synthase (GpgS) captured the Michaelis complex and revealed the signature geometry of front‐side catalysis: short donor C1′‐acceptor O distances and a stabilizing hydrogen bond between the acceptor hydroxyl and the donor *β*‐phosphate that promotes leaving‐group departure (Albesa‐Jové et al., 2015). Consistently, curated comparisons of retaining GTs (including modeled LgtC and solved GalNAc‐T2 complexes) highlight near‐identical donor–acceptor geometries and the recurring *β*‐phosphate–acceptor H‐bond motif (Albesa‐Jové et al., 2015). Building on this, a trends review summarized the then‐emerging consensus that most retaining GTs favor an SNi‐type pathway stabilized by electrostatics around the oxocarbenium‐like center (Ardevol et al., 2016).

The GT6 *α*1,3‐galactosyltransferase (*α*3GalT) family has been a focal point because it uniquely places a conserved acidic residue near the anomeric center, seemingly poised to act as a nucleophile. Early metadynamics/QM/MM work on a GT6 enzyme predicted the formation of a covalent glutamate–galactose adduct consistent with a double‐displacement route (Rojas‐Cervellera et al., 2013). However, subsequent crystallographic trapping of native ternary complexes of α3GalT with UDP‐Gal and lactose (and related analogs) instead supported a substrate‐assisted SNi‐type mechanism, again emphasizing the organizing *β*‐phosphate–acceptor H‐bond and placing the conserved Glu in roles of acceptor positioning/transition‐state stabilization rather than nucleophilic attack; the authors noted that a double‐displacement path would require additional conformational rearrangements not observed in the Michaelis complex (Albesa‐Jové et al., 2017).

Importantly, recent work has also shown that double‐displacement is possible in at least some retaining GTs with unique substrates and folds. Forrester and co‐workers demonstrated that the retaining *β*‐Kdo transferase WbbB (GT99) forms a covalent Asp232–Kdo adduct, captured by MS and x‐ray crystallography, and that catalysis proceeds via two inverting steps separated by a rearrangement of the enzyme‐linked adduct into a second sub‐site before transfer to the acceptor (Forrester et al., 2022). This architecture, as supported by computational studies (Sagiroglugil et al., 2024), decouples leaving‐group stabilization from acceptor activation and appears necessary to accommodate the bulky, anomeric‐carboxylated ulosonic acid donor. In this context, *α*3GalT, a model enzyme for the historical double‐displacement proposals and modern structural evidence for SNi‐like chemistry, is a useful system for analyzing how residues located around the active site, particularly in the flexible C‐terminus loop, influence enzyme function.

In the present work, we have investigated the role of amino acids in the C‐terminal region in catalysis and stability through alanine mutagenesis. Additionally, mutants D225A, from the DXD motif, and E317A, previously proposed as a potential nucleophile, were characterized for stability. For all mutants, the apparent kinetic parameters for both donor and acceptor substrates were determined. Stability was studied with respect to the stabilizing effect of the donor substrate (UDP‐Gal) or UDP in the presence of a denaturing agent. Mutants of K359A, Y361A, R365A, and V363A of the C‐terminus abolished enzyme activity. These same mutants showed the lowest stabilization with the donor or UDP, suggesting that these amino acids interact directly with the substrate. The same results were found for the inactive mutants D225A and E317A. These results highlight the role of the C‐terminus conformational change in building up the catalytic site.

## 2 MATERIALS AND METHODS

### 2.1 Bacterial strains and culture media

*Escherichia coli* DH5*α* (F^−^ Φ80d*lac*ZΔM15 Δ(*lac*ZYA‐*arg*F)U169 *deo*R recA1 *hsd*R17(r_K_^−^, m_k_^+^) *pho*A *sup*E44 λ^−^ *thi*‐1 *gyr*A96 *rel*A1) was used for plasmid propagation and transformation with the mutagenic polymerase chain reaction (PCR). *E. coli* BL21(DE3) (F^−^ *omp*T *hsd*S_B_ *gal dcm* (DE3)) was used for protein expression. For plasmid isolation, bacteria were grown in 2YT medium, and for protein expression, LB medium supplemented with 0.4 mM IPTG was used. Ampicillin at 100 μg/mL was added when appropriate.

### 2.2 Chemicals and enzymes

Urea (molecular biology‐grade) and UDP‐1‐^3^H‐galactose were purchased from Sigma. Restriction endonucleases were from Boehringer Mannheim, and pfuTurbo DNA polymerase was from New Stratagene. DNA sequencing was performed with the T7 sequencing kit from Pharmacia Biotech Inc. Oligonucleotides were synthesized by Thermo Scientific. All buffers and solutions used for kinetic and urea denaturation experiments were degassed before use.

### 2.3 Site‐directed mutagenesis by PCR

The gene coding for *α*‐1,3‐galactosyl transferase bovine previously cloned from the genomic DNA (Joziasse et al., 1989) and subcloned into pET15b as a 932 bases *NdeI*/*BamHI* fragment codifying for the catalytic domain of this enzyme (Monegal, Pinyol, & Planas, 2005) was used as the template for mutagenic PCR following the QuikChange method of Stratagene. The mutagenic primers were as follows (mismatches are in boldface):

T358A, 5′‐GTCTTGGCAG**A**CAAAAGAGTATA‐3′;

K359A, 5′‐GTCTTGGCAGACA**AAA**GAGTATAATGTGGTT‐3′;

E360A, 5′‐GTCTTGGCAGACAAAAG**AG**TATAATGTGGTT‐3′;

Y361A, 5′‐GGCAGACAAAAGAG**TA**TAATGTGGTTAGAAAT‐3′;

N362A, 5′‐GGCAGACAAAAGAGTAT**AAT**GTGGTTAGAAAT‐3′;

V363A, 5′‐GGCAGACAAAAGAGTATAATG**T**GGTTAGAAATAATGTCTG‐3′;

V364A, 5′‐ATAATGTGG**TT**AGAAATAATGTCTG‐3′;

R365A, 5′‐ATAATGTGGTT**AG**AAATAATGTCTG‐3′;

N366A, 5′‐AATGTGGTTAGA**AA**TAATGTCTG‐3′;

N367A, 5′‐GGTTAGAAAT**AA**TGTCTGACTTTGGG‐3′;

V368A, 5′‐GGTTAGAAATAATG**TC**TGACTTTGGG‐3′.

And their respective complementary oligonucleotides. Positive clones were confirmed by complete sequencing of the entire gene.

### 2.4 Protein expression and purification of wild‐type and mutant enzymes

*E. coli* BL21(DE3)‐transformed cells were grown in LB medium (1 L) at 37°C, orbital shaking at 250 rpm for 10 h, induced with 0.4 mM IPTG, and then grown for an additional 4 h at 30°C. Cultures were harvested by centrifugation, and the cell pellet was resuspended in 20 mM Tris, 0.5 M NaCl, pH 7.9, with 1 mM PMSF as a protease inhibitor, then sonicated to isolate the soluble intracellular fraction. The crude lysate was purified by affinity chromatography using a 5 mL HiTrap column charged with Ni^2+^. *α*3GalT was eluted using an imidazole gradient (0–0.25 M) and analyzed by SDS–PAGE. The enzyme was quantified spectrophotometrically by measuring its absorbance at 280 nm, with a calculated extinction coefficient (ε) of 70,410 M^−1^ cm^−1^.

### 2.5 Enzyme assay and kinetics

Steady‐state kinetic studies were carried out as described previously (Monegal, Bulone, & Planas, 2005) using a radiochemical assay. The reaction mixture contained variable concentrations of UDP‐Gal and lactose, 13 mM MnCl_2_, 0.13 mg/mL BSA, 50 mM KCl, 13 mM HEPES, pH 7.0, and the enzyme. Wild‐type and mutant activity were measured at varying concentrations of lactose (0.5–15 mM, acceptor substrate) for a saturating concentration of UDP‐[^3^H] galactose (50 μM, donor substrate) and vice versa (8–250 μM donor at 10 mM acceptor), and enzyme concentration at 3.7 nM. All experiments were performed in triplicate, and the average with its respective standard deviation was plotted for further analysis. The data were analyzed by fitting to the equations:

(1) $$v_{\mathrm{UDP}-\mathrm{Gal}}=\frac{k_{\text{catUDP}-\mathrm{Gal}}^{\mathrm{app}}\left[\mathrm{UDP}-\mathrm{Gal}\right]}{K_{\text{mUDP}-\mathrm{Gal}}^{\mathrm{app}}+\left[\mathrm{UDP}-\mathrm{Gal}\right]}$$

(2) $$v_{\mathrm{Lac}}=\frac{k_{\text{catLac}}^{\mathrm{app}}\left[\mathrm{Lac}\right]}{K_{\text{mLac}}^{\mathrm{app}}+\left[\mathrm{Lac}\right]}$$

(3) $$v_{\mathrm{Lac}}=\frac{k_{\text{catLac}}^{\mathrm{app}}\left[\mathrm{Lac}\right]}{K_{\text{mLac}}^{\mathrm{app}}+\left[\mathrm{Lac}\right]+\frac{{\left[\mathrm{Lac}\right]}^{2}}{K_{\text{iLac}}^{\mathrm{app}}}}$$

where $K_{m}^{\mathrm{app}}$, $k_{\mathrm{cat}}^{\mathrm{app}}$ are the kinetic apparent constants for both acceptor and donor substrates; and $k_{i}^{\mathrm{app}}$ is an apparent constant of inhibition for the acceptor in Equation (3).

### 2.6 Equilibrium urea denaturation

Unfolding was monitored by fluorescence spectroscopy in a FluoroMax‐2 spectrofluorimeter with excitation at 281 nm (2‐nm slit) and the emission spectra being recorded from 300 to 450 nm (3‐nm slit) in thermostatted cuvette holders at 30°C. For each data point collected, wt or mutant *α*‐1,3‐galactosyltransferases in HEPES/KCl buffer (pH 7) and 13 mM MnCl_2_ were diluted to 20 μg/mL in a degassed urea solution in the same buffer and incubated for 12 h at 30°C. To study the stabilizing effect of UDP‐Gal or UDP, 50 μM of each was added to the urea solutions containing the wt enzyme or mutants and incubated under the same conditions described above. All experiments were performed in triplicate, and the average with respective standard deviations was plotted for further analyses.

The data were analyzed as described previously for *β*‐glucanase mutants (Pons et al., 1995, 1997) using Equation (4):

(4) $$F=\frac{\left(\alpha_{N}+\beta_{N}\left[D\right]\right)+\left(\alpha_{U}+\beta_{U}\left[D\right]\right)\exp\left\{\left(m\left[D\right]-{\left[D\right]}_{50\%}\right)/\mathrm{RT}\right\}}{1+\exp\left\{\left(m\left[D\right]-{\left[D\right]}_{50\%}/\mathrm{RT}\right)\right\}}$$

where F is the measured fluorescence, $\alpha_{N}$ and $\alpha_{U}$ are the intercepts and $\beta_{N}$ and $\beta_{U}$ are the slops of the base line at low (*F*) and high (*U*) denaturant concentrations, [*D*] is the denaturant (urea) concentration, [*D*]_50%_ is the concentration of denaturant at which 50% of the protein is unfolded, and *m* is the slop of transition. The free energy of unfolding in the absence of denaturant ($\Delta {G_{U}}^{H_{2}O}$) is then calculated with Equation (5):

(5) $$\Delta {G_{U}}^{H_{2}O}=m{\left[D\right]}_{50\%}$$

Since individual *m* values for each mutant are subjected to significant standard errors, we used the corresponding $m_{\mathrm{av}}$ value to calculate the free energies of unfolding in the absence of denaturant for the wt and mutants with UDP and without UDP, respectively. Then, Equation (2) becomes Equation (6):

(6) $$\Delta {G_{U}}^{H_{2}O}=m_{\mathrm{av}}{\left[D\right]}_{50\%}$$

The difference in stability between two enzymes is evaluated as shown in Equation (7):

(7) $$\Delta \Delta {G_{U}}^{H_{2}O}=\Delta {G_{U}}^{H_{2}O}\left(a\right)-\Delta {G_{U}}^{H_{2}O}\left(b\right)$$

where a and *b* are the mutant and wt enzymes, respectively, in their free or UDP‐complex forms.

### 2.7 Molecular modeling

A full‐length catalytic domain (including the C‐terminus) model of the apo catalytic domain (Glu80–Val368) of α3GalT was created using AlphaFold 3 (Abramson et al., 2024). To predict substrate‐enzyme interactions, a ternary model of *α*3GalT/UDP‐Gal/Lac was assembled. Using YASARA v.25.12.1 (Ozvoldik et al., 2023), the atomic coordinates of UDP‐Gal, Lac, including a water oxygen and Co^2+^ ion, were transferred from the crystal structure in the Protein Data Bank (PDB: 5NRD) to the modeled α3GalT. The Co^2+^ was replaced with Mn^2+^. The resulting complex was energy‐minimized using the AMBER14 force field (Duan et al., 2003) with explicit TIP3P water molecules as the solvent (Jorgensen et al., 1983). The system was placed in a cubic simulation box extending 10 Å from all atoms, and periodic boundary conditions were applied. The optimized complex was further refined with short molecular dynamics simulations as described previously (de Groot et al., 1997).

### 2.8 Molecular dynamics simulations

Refined structures (see Molecular Modeling) of both apo *α*3GalT and the ternary complex were each subjected to molecular dynamics simulations carried out in YASARA v.25.12.1 (Ozvoldik et al., 2023). Each system, apo *α*3GalT and the *α*3GalT/UDP‐Gal/lactose ternary complex, was simulated in three independent replicas. The replicas were generated from the same minimized and equilibrated starting structure but with different initial velocity distributions/random seeds. Each system was simulated in an aqueous solution containing 0.154 M Na^+^ and Cl^−^ ions at pH 7.0. The simulations were performed using the AMBER14 force field (Duan et al., 2003). Ligands were parameterized using the AMBER14 force field, while the metal ion was represented using the standard AMBER14 ion parameters implemented in YASARA. Periodic boundary conditions were used. Long‐range electrostatic interactions were treated using the particle‐mesh Ewald method, and short‐range non‐bonded interactions were calculated using an 8 Å cutoff. The systems were solvated in a cubic simulation box extending 10 Å from all atoms, ensuring adequate solvent (TIP3P water) padding and avoiding artefactual interactions between periodic images. Prior to the production simulations, each system was energy‐minimized and equilibrated. Temperature was maintained using the YASARA rescaling thermostat (Berendsen et al., 1984), and pressure was controlled using solvent‐density‐based pressure control to maintain the correct water density. The simulations were run for 200 ns.

The root‐mean‐square deviations (RMSDs) of C*α* and the per‐residue root‐mean‐square fluctuation (RMSF) were analyzed throughout the MD trajectory. Graphical analysis was performed in PyMOL Molecular Graphics System, Version 1.20, Schrödinger, LLC.

Binding energies for UDP‐Gal and lactose were calculated from the MD trajectories using the YASARA binding‐energy analysis macro. For each trajectory snapshot, the binding energy ($E_{\text{bind}}$) was estimated from the potential ($E_{\mathrm{pot}}$) and solvation ($E_{\text{solv}}$) energies of the separated receptor ($E^{\mathrm{rec}}$), ligand ($E^{\mathrm{lig}}$), and complex ($E^{\mathrm{cmp}}$):

(8) $$E_{\text{bind}}=\left(E_{\mathrm{pot}}^{\mathrm{rec}}+E_{\text{solv}}^{\mathrm{rec}}\right)+\left(E_{\mathrm{pot}}^{\mathrm{lig}}+E_{\text{solv}}^{\mathrm{lig}}\right)-\left(E_{\mathrm{pot}}^{\mathrm{cmp}}+E_{\text{solv}}^{\mathrm{cmp}}\right)$$

Solvation energies were calculated using the BoundaryFast method implemented in YASARA. The reported trajectory averages from the YASARA binding‐energy macro are potential/solvation‐energy estimates and were used only for qualitative comparison of ligand‐associated energy profiles, not as absolute thermodynamic binding free energies. These estimates indicate that the donor and acceptor experience different interaction environments in the ternary complex.

## 3 RESULTS

### 3.1 Molecular modeling

**FIGURE 2:**
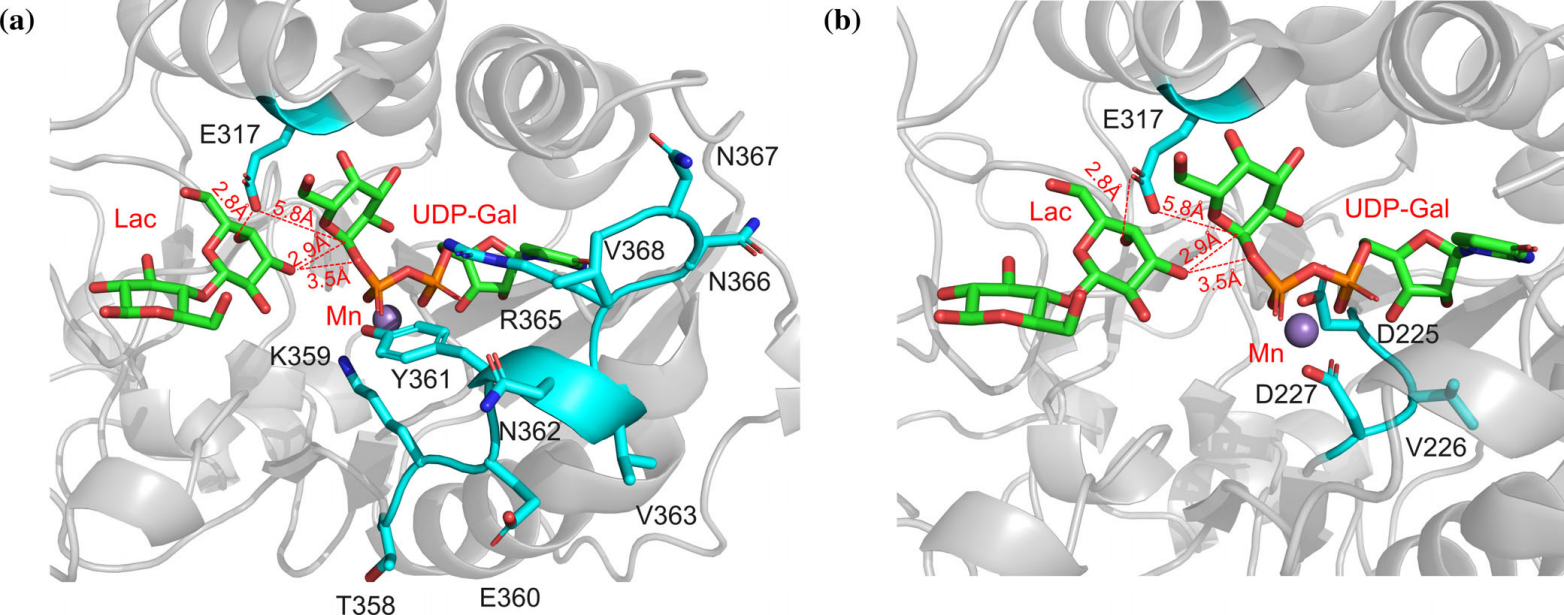
Structural basis of C‐terminus loop function in *α*3GalT. (a) Structural model of bovine *α*3GalT bound to UDP‐Gal and lactose. The flexible C‐terminus loop (residues 358–368) contributes key side chains (K359, Y361, R365) that directly contact the donor. Additional residues (V363, V364, N366–V368) support loop conformation and packing. (b) Active‐site view showing the DVD motif (D225–D227) coordinating Mn^2+^ and bridging the donor phosphate, with E317 positioned near the acceptor.

The ligand‐induced stabilization was previously observed in a crystallographic study of *α*3GT mutant R365L binding to a non‐hydrolyzable analog, UDP‐2F‐Gal (PDB 2JCF; corrected to 2VFZ) (Jamaluddin et al., 2007), and later in the ternary Michaelis complex containing the donor and acceptor (PDB: 5NRD) (Albesa‐Jové et al., 2017). However, both structures lack the modeled C‐terminus because these residues were disordered in the crystallized structures: 2VFZ, solved as a dimer, is modeled until Lys259 in chain A and at Val363 in chain B, while 5NRD, also solved as a dimer, is modeled until Arg365 in chain A and Thr358 in chain B. In the present work, the AlphaFold 3‐modeled apo‐structure showed a complete C‐terminus conformation, consistent with the *α*7 helix previously reported in the crystal structure of *α*3GT with UDP as the ligand (PDB: 1K4V) (Boix et al., 2001), with an RMSD of 0.154 Å of C*α* (269 atoms), indicating that the model is almost identical to the crystal structure (Figure S1). Thus, the modeled C‐terminal conformation should be viewed as a simulation starting point consistent with available crystallographic evidence, while the functional relevance of this region is supported independently by the alanine‐scanning, kinetic, and ligand‐dependent unfolding experiments described below. Therefore, the model was used to build the ternary complex, in which the carboxylate of Glu317 is 5.8 Å from the C1 of UDP‐Gal, while C1 is 2.9 Å from O3 of Lac, as was obtained by MD simulations (discussed later) (Figure 2).

### 3.2 Enzyme expression and purification

Point mutations to alanine in the C‐terminus loop residues from Thr358 to Val368 (Figure 2) were prepared by site‐directed mutagenesis by PCR. The mutant and wt proteins were purified to 95% purity, as judged by SDS‐PAGE. Expression and purification yields varied with the mutant, ranging from 3 to 35 mg/L. The lowest yield was observed for the K359A mutant, whereas most others were similar to the wt.

### 3.3 Catalytic parameters of wild‐type and mutant enzymes

Apparent kinetic constants, $k_{\mathrm{cat}}^{\mathrm{app}}$ and $K_{m}^{\mathrm{app}}$, for wt and mutants (C‐terminus loop, T358A to V368A) were determined with specific substrates, both donor (UDP‐Gal) and acceptor (lactose), in ranges from 0.4 to 250 μM, and from 0.5 to 10 mM, respectively. All reactions were performed under the same conditions: 30°C, HK buffer (13 mM HEPES, 50 mM KCl) pH 7, 0.13 mg/mL BSA, and 13 mM MnCl_2_. The formation of the product was monitored by a radiometric assay using UDP‐1‐^3^H‐Gal as donor substrate (Monegal, Bulone, & Planas, 2005). Under these conditions, all the proteins studied exhibited a linear progress curve during the initial 20 min of the reaction. Four of the mutants' activities were not detected: K359A, Y361A, V363A, and R365A. The mutants N367A and V368A exhibit two‐ and three‐fold greater catalytic efficiency (*k*_cat_/*K*_M_) than the wt with respect to the donor substrate, while N362A and V364A show 51% and 12% efficiency, respectively (Table 1). With respect to the acceptor substrate, substrate inhibition was observed in almost all mutants, except for T358A and the wt. The efficiencies were similar to those of the wt, except for N366A, which showed a twofold increase, and N362A, which showed nearly a 60% increase; however, V364A did not reach saturation within the range of donor‐substrate concentrations studied (Table 2).

**TABLE 1:** Kinetic parameters (apparent constants) for the donor substrate UDP‐Gal in wt and mutants of *α*‐1,3‐galactosyltransferase at a saturating acceptor (lactose) concentration.

Variant	$k_{\mathrm{cat}}^{\mathrm{app}}$ (s^−1^)	$K_{m}^{\mathrm{app}}$ (μM)	kcatappKmapp (s^−1^ μM^−1^)	kcatappKmapp (%)
WT	1.6 ± 0.03	8.1 ± 0.5	0.2 ± 0.02	100
T358A	1.5 ± 0.03	4.4 ± 0.5	0.4 ± 0.04	178
K359A	Activity not detected			0.0
E360A	2.3 ± 0.05	9.4 ± 0.9	0.2 ± 0.03	120
Y361A	Activity not detected			0.0
N362A	0.9 ± 0.03	9.1 ± 1.5	0.1 ± 0.02	51
V363A	Activity not detected			0.0
V364A	0.2 ± 0.003	9.0 ± 0.5	0.03 ± 0.002	12
R365A	Activity not detected			0.0
N366A	1.6 ± 0.01	6.7 ± 0.3	0.2 ± 0.01	121
N367A	1.5 ± 0.01	2.7 ± 0.5	0.5 ± 0.06	276
V368A	2.1 ± 0.04	2.9 ± 0.5	0.7 ± 0.1	374

*Note*: Conditions: 13 mM HEPES, pH 7.0, 50 mM KCl, 13 mM MnCl2, 0.13 mg/mL BSA, 30°C. [donor] = 0.4–250 μM, [acceptor] = 10 mM, [enzyme] = 3.7 nM. Full data in Figure S2.

**TABLE 2:** Kinetic parameters (apparent constants) belonging to acceptor substrate lactose for wt and mutants of *α*‐1,3‐galactosyltransferase at saturating donor substrate (UDP‐Gal) concentration.

Variant	$k_{\mathrm{cat}}^{\mathrm{app}}$ (s^−1^)	$K_{m}^{\mathrm{app}}$ (mM)	kcatappKmapp (s^*−1*^ mM^−1^)	kcatappKmapp (%)	$K_{\text{iLac}}^{\mathrm{app}}$ (mM)
WT	0.9 ± 0.02	0.7 ± 0.06	1.4 ± 0.2	100	
T358A	1.5 ± 0.04	0.9 ± 0.1	1.7 ± 0.2	120	
K359A	Activity not detected				
E360A	2.4 ± 0.3	1.7 ± 0.4	1.4 ± 0.4	101	21.4 ± 6.3^a^
Y361A	Activity not detected				
N362A	1.1 ± 0.1	1.2 ± 0.3	0.9 ± 0.3	64	21.6 ± 6.6^a^
V363A	Activity not detected				
V364A	>0.6 ± 0.1	>19.3 ± 5.6^b^	0.03 ± 0.02	2	
R365A	Activity not detected				
N366A	2.1 ± 0.1	0.7 ± 0.1	2.8 ± 0.6	202	43 ± 12^a^
N367A	2.3 ± 0.4	1.6 ± 0.7	1.4 ± 0.9	103	50 ± 47^a^
V368A	1.9 ± 0.2	0.9 ± 0.2	2.1 ± 0.7	152	117 ± 110^a^

*Note*: Conditions: 13 mM HEPES, pH 7.0, 50 mM KCl, 13 mM MnCl_2_, 0.13 mg/mL BSA, 30°C. [acceptor] = 0.5–10 mM, [donor] = 250 μM, [enzyme] = 3.7 nM. Full data in Figure S3.

^a^ Have shown substrate inhibition kinetics.

^b^ Has not reached saturation.

### 3.4 Stability by equilibrium urea denaturation

**FIGURE 3:**
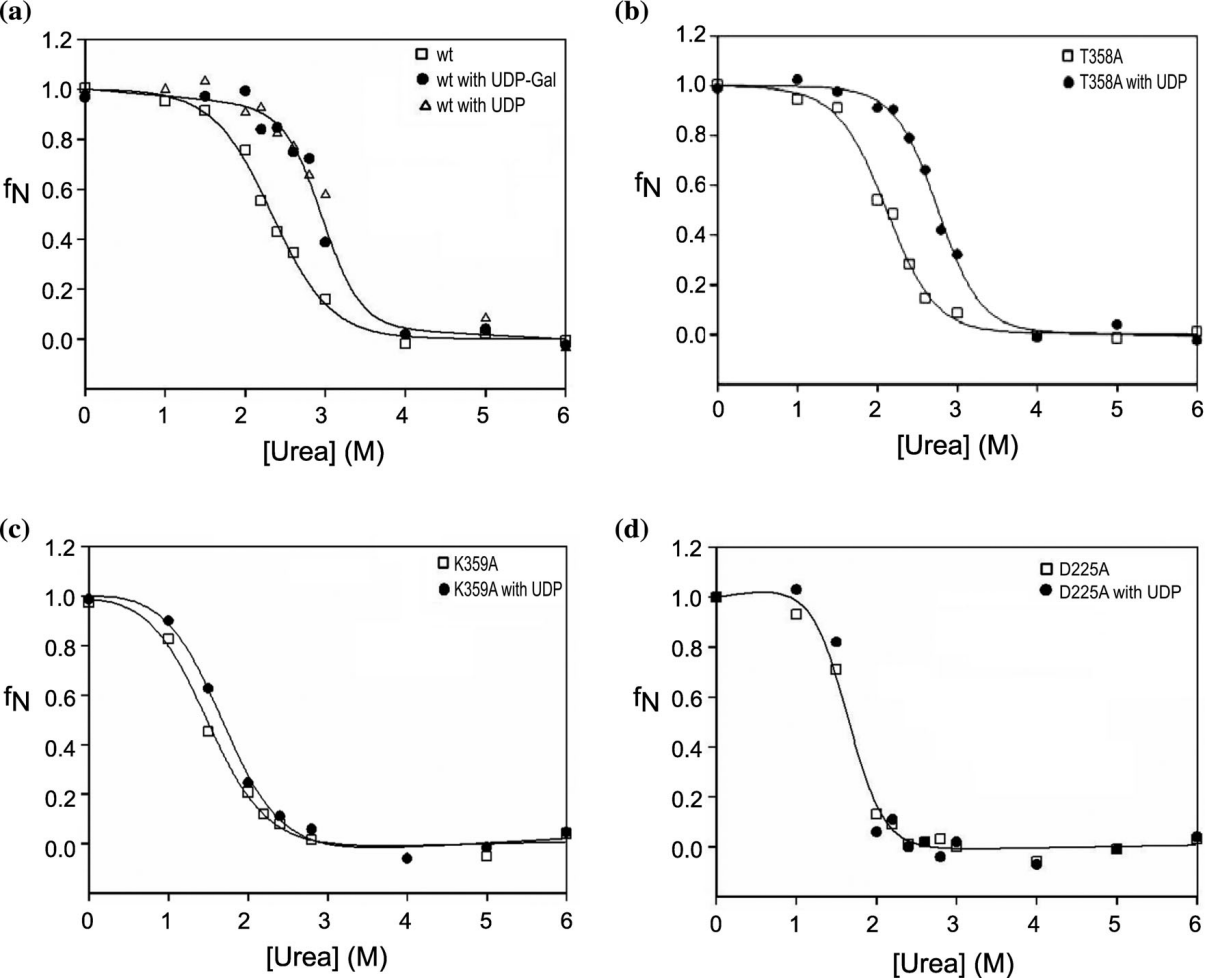
Urea‐induced unfolding of α3GalT wt and selected mutants in the presence and absence of UDP/UDP‐Gal. (a) The wt enzyme shows a cooperative unfolding transition at 2.3 M urea. The addition of UDP or UDP‐Gal shifts the transition to a higher denaturant concentration (3.1 M), indicating significant ligand‐induced stabilization. (b) T358A mutant shows ligand‐induced stabilization similar to that of the wt. (c). K359A mutant shows a significant loss of ligand‐induced stabilization compared to the wt. (d) D225A mutant (DVD motif) is destabilized and fails to gain stability from UDP, reflecting the importance of Asp225 in coordinating Mn^2+^ for phosphate binding. Plots for the rest of the mutants are shown in Figure S4.

The stability of the enzymes reported in this study was examined by urea denaturation, assuming a two‐state transition using the model of Clarke and Fersht (Clarke & Fersht, 1993). Unfolding was monitored by measuring the dependence of fluorescence intensity on urea concentration (Figures 3 and S4). The data were analyzed using Equations ((4), (5), (6), (7))–((4), (5), (6), (7)) in Material and Methods. The calculated values of m and [D]_50%_ are presented in Table 3. The stability of wt and mutant enzymes is calculated according to Equation (6) and presented in Table 4. Most of the mutants show some destabilization of the free enzyme compared to the wt, except mutants K359A and Y361A, which show a larger destabilization. The energetic contribution of the UDP ligand to stability was calculated using Equation (7), in which a and *b* are the enzymes with and without UDP, respectively. The estimated values in Table 4 display a stabilizing effect for UDP ligand in the wt (same effect for UDP‐Gal and UDP, Figure 3a). Some of the mutants also show stabilizing effect by UDP as the wt (Figure 3b), whereas those involved in interactions with UDP exhibit a decrease in the stabilization effect (R365A, Y361A, and K359A, Figure 3c), which is almost nonexistent for D225A (Figure 3d). These unfolding profiles demonstrate that UDP/UDP‐Gal stabilizes wt *α*3GalT by ordering the C‐terminus loop, whereas mutations at residues directly involved in donor, phosphate, or metal binding abolish this effect.

**TABLE 3:** Equilibrium urea denaturation data for wt and mutants of *α*‐1,3‐galactosyltransferase with and without UDP.

Variant	Without UDP		With UDP	
	m (Jmol^−2^)	[D]_50%_ (M)	m (Jmol^−2^)	[D]_50%_ (M)
wt	6828 ± 883	2.3 ± 0.07	6686 ± 1050	3.1 ± 0.06
T358A	8055 ± 1899	2.1 ± 0.09	9207 ± 1380	2.8 ± 0.05
K359A	7200 ± 1236	1.5 ± 0.07	7876 ± 1068	1.7 ± 0.05
E360A	13,052 ± 2592	2.4 ± 0.05	11,192 ± 2702	2.9 ± 0.06
Y361A	5618 ± 442	1.2 ± 0.05	5282 ± 329	1.6 ± 0.05
N362A	10,447 ± 2329	1.6 ± 0.10	8952 ± 767	2.4 ± 0.03
V363A	9008 ± 1318	1.6 ± 0.08	8600 ± 1420	2.5 ± 0.05
V364A	8489 ± 619	1.7 ± 0.03	8832 ± 1098	2.2 ± 0.04
R365A	9436 ± 716	1.8 ± 0.04	11,180 ± 2955	2.3 ± 0.08
N366A	11,271 ± 1420	1.7 ± 0.05	8952 ± 767	2.4 ± 0.03
N367A	14,739 ± 1872	2.2 ± 0.03	9608 ± 1893	2.8 ± 0.06
V368A	14,629 ± 1984	2.4 ± 0.03	17,011 ± 6228	2.9 ± 0.07
D225A	13,925 ± 1273	1.7 ± 0.03	17,812 ± 3836	1.7 ± 0.05
E317A	7503 ± 1118	1.7 ± 0.17	15,438 ± 1904	1.6 ± 0.02
Average	10,014		10,473	

**TABLE 4:** Free energies of unfolding for wt and mutants of *α*‐1,3‐galactosyltransferase determined by equilibrium urea denaturation at 30°C.

Variant	No UDP ${∆G}_{U}^{H_{2}O}$(kJ/mol)	With UDP ${∆G}_{U}^{H_{2}O}$(kJ/mol)	${∆∆G}_{U}^{H_{2}O}$(UDP–no UDP) (kJ/mol)
wt	−23.37	−32.02	−8.65
T358A	−21.19	−28.91	−7.72
K359A	−14.85	−17.48	−2.63
E360A	−23.61	−30.82	−7.21
Y361A	−11.69	−16.59	−4.90
N362A	−16.48	−24.94	−8.46
V363A	−16.41	−26.27	−9.87
V364A	−16.62	−23.38	−6.76
R365A	−17.84	−23.98	−6.14
N366A	−16.86	−24.94	−8.08
N367A	−22.42	−29.41	−6.99
V368A	−24.10	−30.67	−6.57
D225A	−17.12	−17.45	−0.33
E317A	−16.93	−16.81	+0.12

### 3.5 Molecular dynamics

**FIGURE 4:**
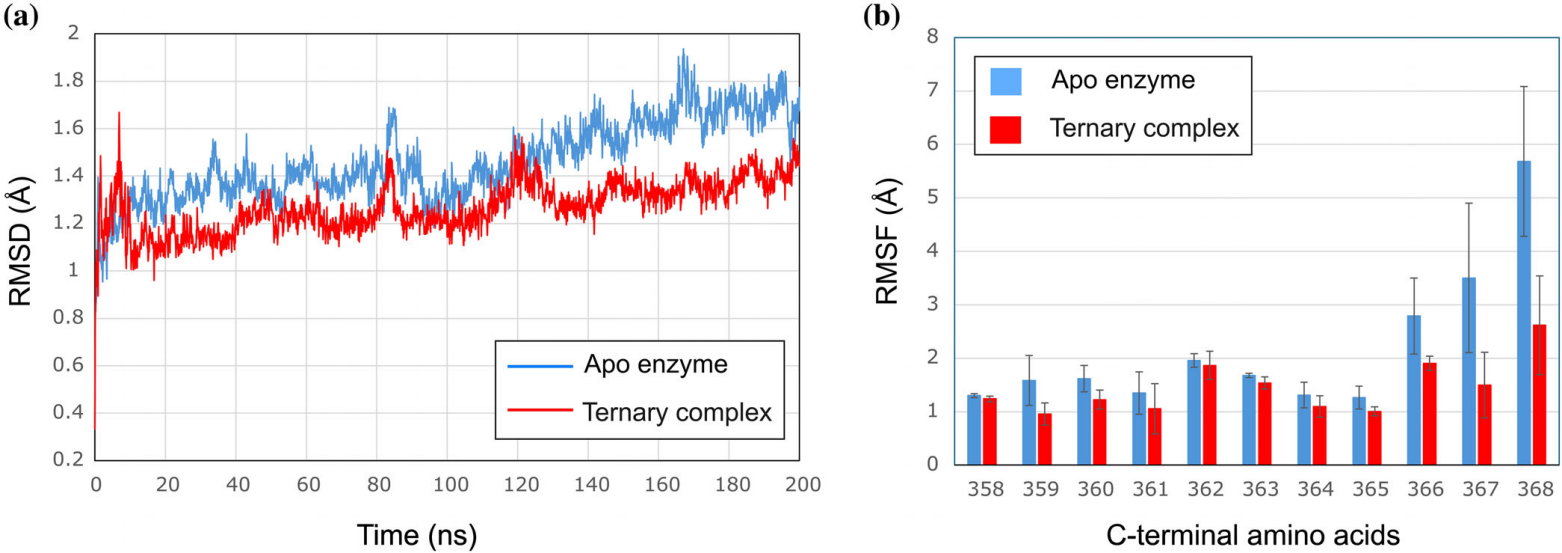
Molecular dynamics analyses of apo *α*3GalT and the ternary complex. All simulations were performed in triplicate (data are shown as the mean of three independent replicas), using the AMBER14 force field at 30°C, pH 7.0, for 200 ns. (a) RMSDs of C*α*. (b) RMSFs per residue in the C‐terminal region 58–368 (error bars represent the standard deviation across replicas).

The MD simulations presented in this work are consistent with crystallographic studies in the literature and with our experimental results (Figure S5). Both the *α*3GalT free form and the α3GalT/UDP‐Gal/Lac ternary complex remain stable over 200 ns. However, the complex exhibited a lower, more stable RMSD of C*α* (~1.2 Å) than the free form (~1.5 Å) (Figure 4a), confirming ligand‐induced conformational tightening, as experimentally supported by equilibrium urea denaturation analysis.

Both experimental and MD simulations have revealed that the dynamics of the C‐terminus loop (residues 358–368) depend on substrate binding. RMSF analysis showed that the average fluctuation of this region decreased from 2.19 Å in the apo enzyme to 1.46 Å in the ternary complex, corresponding to an approximately 33% reduction (Figure 4b). The most significant decreases are observed for the last three C‐terminus residues (366‐368), probably as a consequence of loop stabilization by donor binding through residues Lys359, Tyr361, and Arg365. These residues constitute a phosphate‐binding triad that interacts with the *β*‐ and *α*‐phosphates of UDP‐Gal, as was demonstrated experimentally by inactive alanine mutants. These results indicate that the donor‐binding drives loop stabilization, thereby providing an active‐site conformation suitable for catalysis.

The binding‐energy analysis of MD trajectories showed an asymmetric stabilization pattern between the donor (UDP‐Gal) and the acceptor (Lac). UDP‐Gal showed an average energy estimate of −589 ± 21 kJ/mol, whereas lactose showed an average value of −178 ± 4 kJ/mol. These results are consistent with previous thermodynamic studies using isothermal titration calorimetry (ITC), which revealed stronger binding in the complex α3GT/Mn^2+^/UDP‐Gal than in the *α*3GT/Mn^2+^/UDP/Lac complex, with Δ*G* values of −5.86 kcal/mol and −3.57 kcal/mol, respectively (Boix et al., 2002). This energetic asymmetry is attributed primarily to electrostatic and hydrogen‐bond interactions between UDP‐Gal and Lys359, Tyr361, Arg365, and the DXD motif (D225–D227). The mutational analysis supports the functional importance of these donor/phosphate‐contacting residues. In contrast, lactose appears to be stabilized by fewer and more transient hydrogen‐bond interactions.

The distances between key catalytic atoms were measured during the MD simulations. The distance between the donor's anomeric carbon (C1 of UDP‐Gal) and the carboxylate's oxygen of Glu317 remained above 3.8 Å throughout the trajectory, indicating the unlikely formation of a covalent intermediate. Conversely, the distance between C1 and the acceptor's O3‐hydroxyl of lactose was stable at a shorter distance of about 2.9–3.1 Å, consistent with a pre‐reactive alignment for in‐line front‐side nucleophilic attack.

## 4 DISCUSSION

The x‐ray structures of *α*GalT have shown that the C‐terminus residues are disordered in the free enzyme but adopt a close conformation in only two enzyme/ligand complexes. However, the functional roles of the C‐terminal residues have been poorly investigated. Here, alanine‐scanning mutagenesis was used to analyze the role of the flexible C‐terminus loop (Thr358–Val368) in catalysis, stability, and ligand‐induced stabilization, alongside two benchmark mutations: D225A in the DVD motif (Zhang et al., 2001) and E317A in the catalytic site (Gastinel, 2001; Monegal & Planas, 2006). These data provide functional insights into how *α*3GalT assembles its Michaelis complex and how specific residues contribute to donor/acceptor positioning.

### 4.1 Stabilizing effect of UDP

Ligand‐dependent stabilization assays show that both UDP and UDP‐Gal stabilize the wt enzyme, with similar effects (Figure 3a). The C‐terminus residues interact with the UDP moiety of the donor, so stabilizing effects were determined with UDP as ligand. Active mutants such as V368A, E360A, N367A, and T358A retain substantial stabilization by UDP, while mutants that disrupt phosphate interactions (R365A, K359A, Y361A) or metal binding (D225A) fail to benefit from UDP stabilization. This is consistent with the crystallographic observations. In the x‐ray structure of the free enzyme, the C‐terminus region is not observed (Gastinel, 2001). In contrast, in the x‐ray structures of ligand complexes, such as α3GalT/UDP (Boix et al., 2001), this region is fully resolved or partially solved, as in *α*3GalT/UDP‐F‐Gal (Jamaluddin et al., 2007) and *α*3GalT/UDP‐Gal/Lac complexes (Albesa‐Jové et al., 2017), suggesting that it is structured and stabilized by donor ligand interactions. Upon C‐terminal loop closing, additional inter‐residue interactions also stabilize the C‐terminal helix. Trp195, Arg365, and Tyr361 form a packed triple, stabilized by extensive van der Waals and cation‐π interactions, and Lys359 further stacks on Tyr361 (Figure S6). This network of interactions is likely essential for stabilizing this motif, and indeed, mutants K359A, Y361A, and R365A show severe loss of stabilization. Importantly, E317A also shows almost no stabilization by UDP, in agreement with the Albesa‐Jové et al. (Albesa‐Jové et al., 2017) ternary complex, placing Glu317 too far from the donor C1 to serve as a nucleophile, but in a hydrogen‐bonding distance to the acceptor O4 and with no direct contact with the donor. Here, MD simulations also show that the distance between the donor's anomeric carbon (C1 of UDP‐Gal) and the carboxylate's oxygen of Glu317 remained above 3.8 Å along the simulation. Thus, the E317A mutant reflects a loss of acceptor binding. These findings complement the structural evidence that the *β*‐phosphate of UDP functions as the general base in a substrate‐assisted SNi‐type mechanism, while the C‐terminus residues help orient the donor for catalysis.

### 4.2 Effects on enzyme kinetics

**FIGURE 5:**
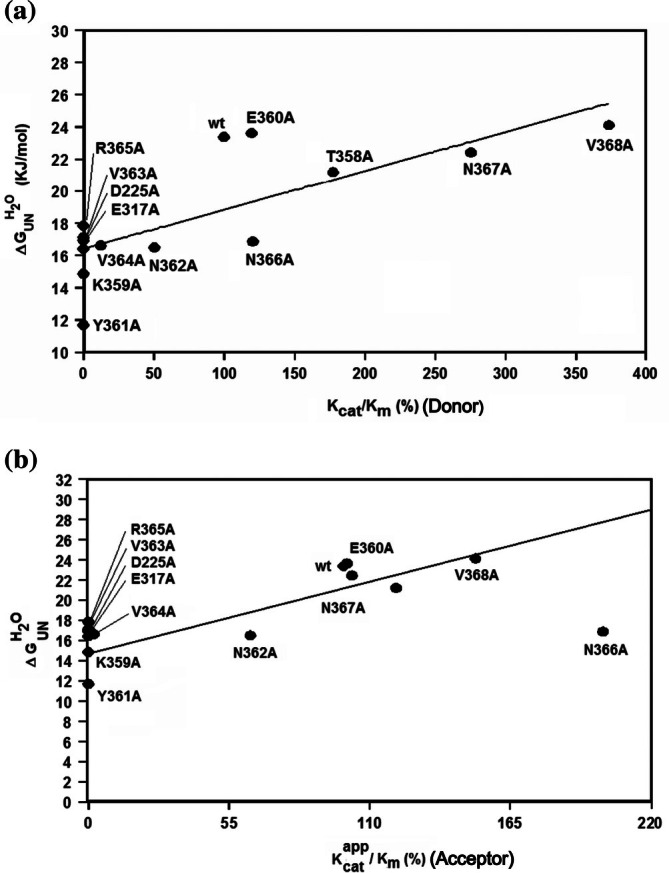
Correlation between catalytic efficiency and conformational stability of *α*3GalT mutants. (a) Catalytic efficiency (*k*_cat_/*K*_*m*_^app^) with UDP‐Gal as donor substrate plotted against unfolding free energy (${∆G}_{\mathrm{UN}}^{H_{2}O}$). (b) Catalytic efficiency (*k*_cat_/*K*_*m*_^app^) with lactose as acceptor substrate plotted against ${∆G}_{\mathrm{UN}}^{H_{2}O}$.

A positive correlation is observed in both panels of Figure 5, indicating that reduced stability generally corresponds to a loss of catalytic efficiency. Mutants T358A, E360A, N362A, V364A, N366A, N367A, and V368A cluster along the trend line, consistent with local structural perturbations that proportionally reduce both stability and activity. Notably, N367A and V368A retain higher than wt efficiency, suggesting that the distal tip of the C‐terminus extension may modulate the activity but is not essential for catalysis (Figure 2). In contrast, K359A, Y361A, R365A, D225A, and E317A showed no detectable activity and minimal stabilization, indicating their direct involvement in donor‐acceptor and phosphate‐metal interactions within the active site. D225 binds Mn^2+^ in the DVD motif, required for phosphate binding. K359, Y361, and R365 stabilize the donor and phosphate groups, explaining their critical function. Thus, these results functionally validate the C‐terminus loop as a key determinant of Michaelis complex assembly.

### 4.3 Mechanistic implications

Our results agree with the mechanisms that have been proposed for GT6 enzymes (Gómez et al. 2012, Albesa‐Jové et al. 2015, Ardevol et al. 2016, Albesa‐Jové et al. 2017). The critical C‐terminus residues we identified (Lys359, Tyr361, Arg365) stabilize the *β*‐phosphate donor substrate via hydrogen bonds. On the other hand, Glu317 is repositioned in the Michaelis complex to stabilize the acceptor, where the distance between O4 and the OE carboxylate of Glu317 remains constant at around 2.8 Å throughout the MD trajectory. MD simulations indicate that the C‐terminus loop flexibility (RMSF AA 358–368) is significantly reduced upon donor binding, in agreement with the observed donor‐induced stabilization. Additionally, the Mn^+2^ coordination remains stable and helps donor binding. The ligand‐dependent loop stabilization correlates with catalytic turnover, providing quantitative support for the relationship between structure, C‐terminus flexibility, and function. Glu317, previously considered a putative nucleophile (Monegal & Planas, 2006; Rojas‐Cervellera et al., 2013), is now also interpreted as a residue that contributes to acceptor orientation. On the Michaelis complex, crystallographic evidence (Albesa‐Jové et al., 2017) as well as MD simulations suggest that the carboxylate group of Glu317 would require an additional conformational change to become a nucleophile to form a covalent intermediate in a double displacement mechanism. On the other hand, comparative QM/MM simulations have suggested that both the SNi‐like and covalent intermediate mechanisms may operate concurrently, as similar activation barrier heights have been calculated for both pathways (Gómez et al., 2013). However, the nucleophilic strength of Glu317 is reduced through its interaction with the acceptor, making it unlikely that Glu317 alone can promote the formation of a covalent intermediate. Altogether, the current evidence favors a front‐side SNi‐like mechanism for *α*3GalT. It is important to note that retaining GTs are not intrinsically SNi‐type enzymes. Forrester et al. (Forrester et al., 2022) recently showed that the *β*‐Kdo transferase WbbB (GT99) forms a covalent Asp–Kdo intermediate and proceeds via two inverting steps with rearrangement of the glycosyl–enzyme adduct. Moreover, the protein provides a Glu side chain, which acts as a base instead of the *β*‐phosphate of UDP. Another Kdo transferase, KpsC, which is a distant homologue of WbbB that belongs to family GT107, was also shown by the same authors to proceed by a double displacement mechanism through a covalent intermediate (Doyle et al., 2023). This mechanism reflects the unusual steric and electronic demands of the CMP‐Kdo donor and its active‐site environment. In contrast, the mechanism followed by *α*3GalT is still controversial: crystal structures of ternary complexes (enzyme/UDP‐Gal/lactose) suggest an SNi‐like mechanism because the putative nucleophile Glu317 remains too far from the anomeric carbon, but QM/MM simulations point to similar energy barriers for both double‐displacement and SNi‐like mechanisms. In either case, however, our results conclude that the C‐terminus loop is essential to stabilize the Michaelis complex and provide a competent complex for catalysis.

## 5 CONCLUSION

The alanine scanning mutagenesis of the C‐terminus region of *α*1,3‐galactosyltransferase shows that residues Lys359, Tyr361, and Arg365 are essential for catalysis, reflecting their direct roles in stabilizing donor, acceptor, and phosphate groups within the substrate/enzyme complex. The loss of UDP stabilization in these mutants, as well as in E317A, demonstrates how this flexible loop and Glu317 work together to maintain the geometry needed for catalysis. Along with MD simulations and equilibrium unfolding assays, these findings confirm that ligand binding induces C‐terminus loop ordering, necessary for catalysis in GT6 enzymes.

## AUTHOR CONTRIBUTIONS

**Javier A. Linares‐Pastén:** Conceptualization; data curation; formal analysis; visualization; writing – original draft; methodology; investigation; writing – review and editing; software; validation; resources. **Antoni Planas:** Conceptualization; formal analysis; methodology; investigation; project administration; validation; funding acquisition; resources; writing – review and editing.

## CONFLICT OF INTEREST STATEMENT

The authors declare no conflicts of interest.

## Supporting information


**Figure S1.** (A) AlphaFold3 model of αGalT structure. (B) Overlay of the AlphaFold3 model and the crystal structure with UDP (PDB 1K4V), showing a ribbon representation of the enzyme backbone and side chain amino acids. Co‐crystalized UDP and Mn^+2^ are shown in sticks.
**Figure S2.** Kinetics of αGalT with donor substrate UDP‐Gal at saturating (10 mM) acceptor (lactose) concentration.
**Figure S3.** Kinetics of *α*GalT with acceptor substrate lactose at saturating (250 μM) donor (UDP‐Gal) concentration.
**Figure S4.**. Urea‐induced unfolding of *α*GalT wt and mutants in the absence and presence of UDP ligand (and UDP‐Gal in the wt). Urea‐induced unfolding of *α*GalT wt and mutants in the absence and presence of UDP ligand.
**Figure S5.** Conformational sampling of lactose and UDP‐Gal during MD simulations of the *α*3GalT ternary complex. Superimposed representative ligand conformations extracted from the 200 ns MD simulations. The UDP‐Gal nucleotide moiety oscillates between conformations resembling the activated Michaelis‐complex‐like 5NRD state (PDB: 5NDR) and the 5NRB‐like state (PDB: 5NBR). Carbon atoms of the 5NRD‐like conformation are shown in blue, whereas carbon atoms of the 5NRB‐like conformation are shown in brown.
**Figure S6.** Inter‐residue interactions in the closed conformation of the C‐terminal helix in the Michaelis complex.

## Data Availability

All data is available in the manuscript and Supporting Information.
