# Supplementary material for: Donor‐induced conformational gating and substrate‐assisted catalysis in α ‐1,3‐galactosyltransferase
Source: Protein Sci. 2026 Aug 15;35(9):e70770. doi: 10.1002/pro.70770 (PMC13476921; doi:10.1002/pro.70770)

SUPPORTING INFORMATION

Donor-Induced Conformational Gating and Substrate-Assisted Catalysis in α-1,3-Galactosyltransferase

Javier A. Linares-Pastén [1,*], Antoni Planas [2,3,*]

[1] Biotechnology and Applied Microbiology, Department of Process and Life Sciences Engineering, LTH, Lund University, PO, P.O Box 124, Lund, Sweden.

[2] Laboratory of Biochemistry, Institut Químic de Sarrià, University Ramon Llull, Via Augusta 390, 08017 Barcelona, Spain.

[3] Chemistry section, Royal Academy of Sciences and Arts of Barcelona, La Rambla 115, 08002 Barcelona, Spain

Figure S1. (A) AlphaFold3 model of αGalT structure. (B) Overlay of the AlphaFold3 model and the crystal structure with UDP (PDB 1K4V).

Figure S2. Kinetics of αGalT with donor substrate UDP-Gal at saturating (10 mM) acceptor (lactose) concentration.

Figure S3. Kinetics of αGalT with acceptor substrate lactose at saturating (250 μM) donor (UDP-Gal) concentration.

Figure S4. Urea-induced unfolding of αGalT wt and mutants in the absence and presence of UDP ligand (and UDP-Gal in the wt).

Figure S5. Conformational sampling of lactose and UDP-Gal during MD simulations of the α3GalT ternary complex.

Figure S6. Inter-residue interactions in the closed conformation of the C-terminal helix in the Michaelis complex.

Figure S1. (A) AlphaFold3 model of αGalT structure. (B) Overlay of the AlphaFold3 model and the crystal structure with UDP (PDB 1K4V), showing a ribbon representation of the enzyme backbone and side chain amino acids. Co-crystalized UDP and Mn^{+2} are shown in sticks.

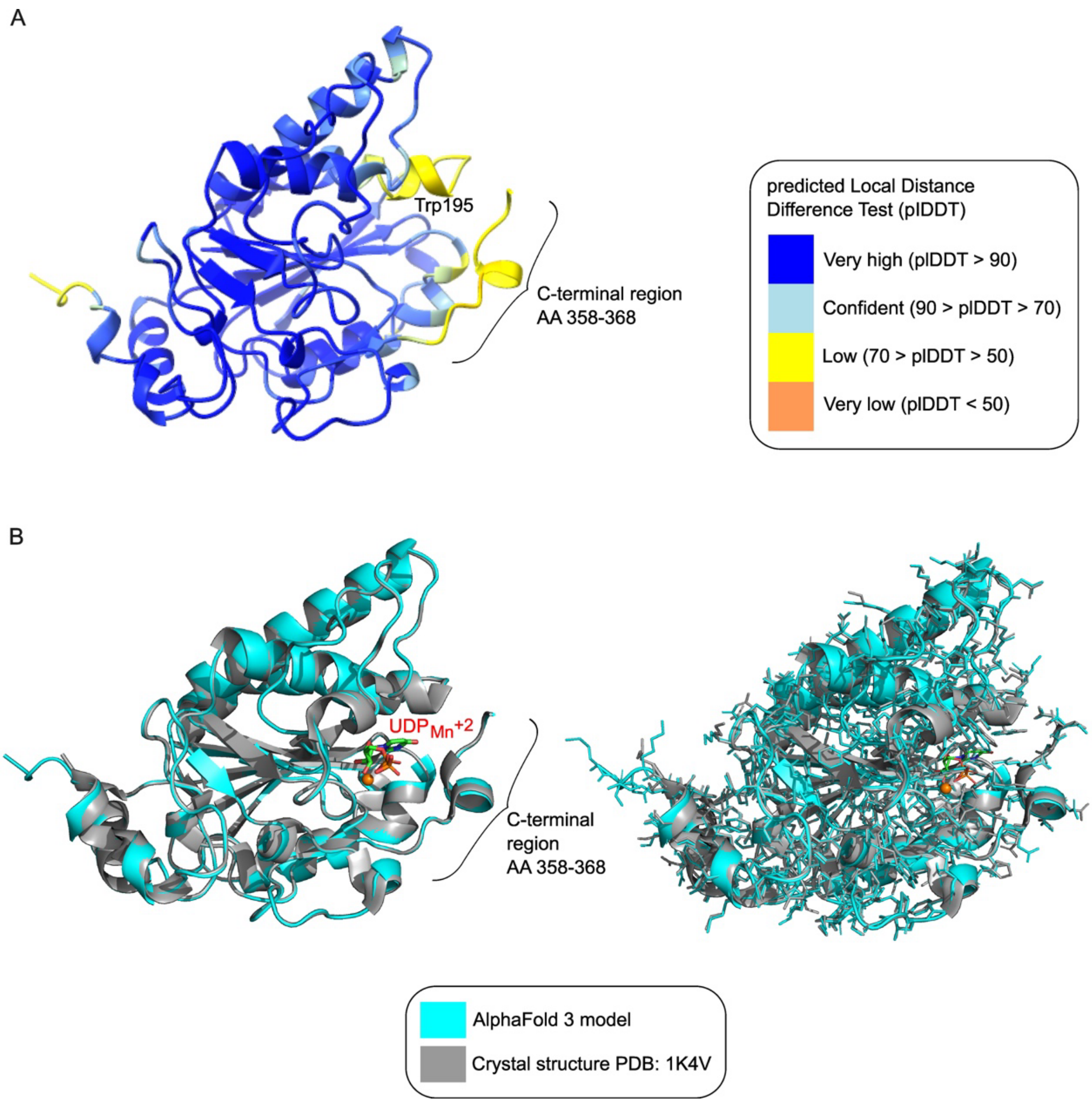

Figure S2. Kinetics of αGalT with donor substrate UDP-Gal at saturating (10 mM) acceptor (lactose) concentration.

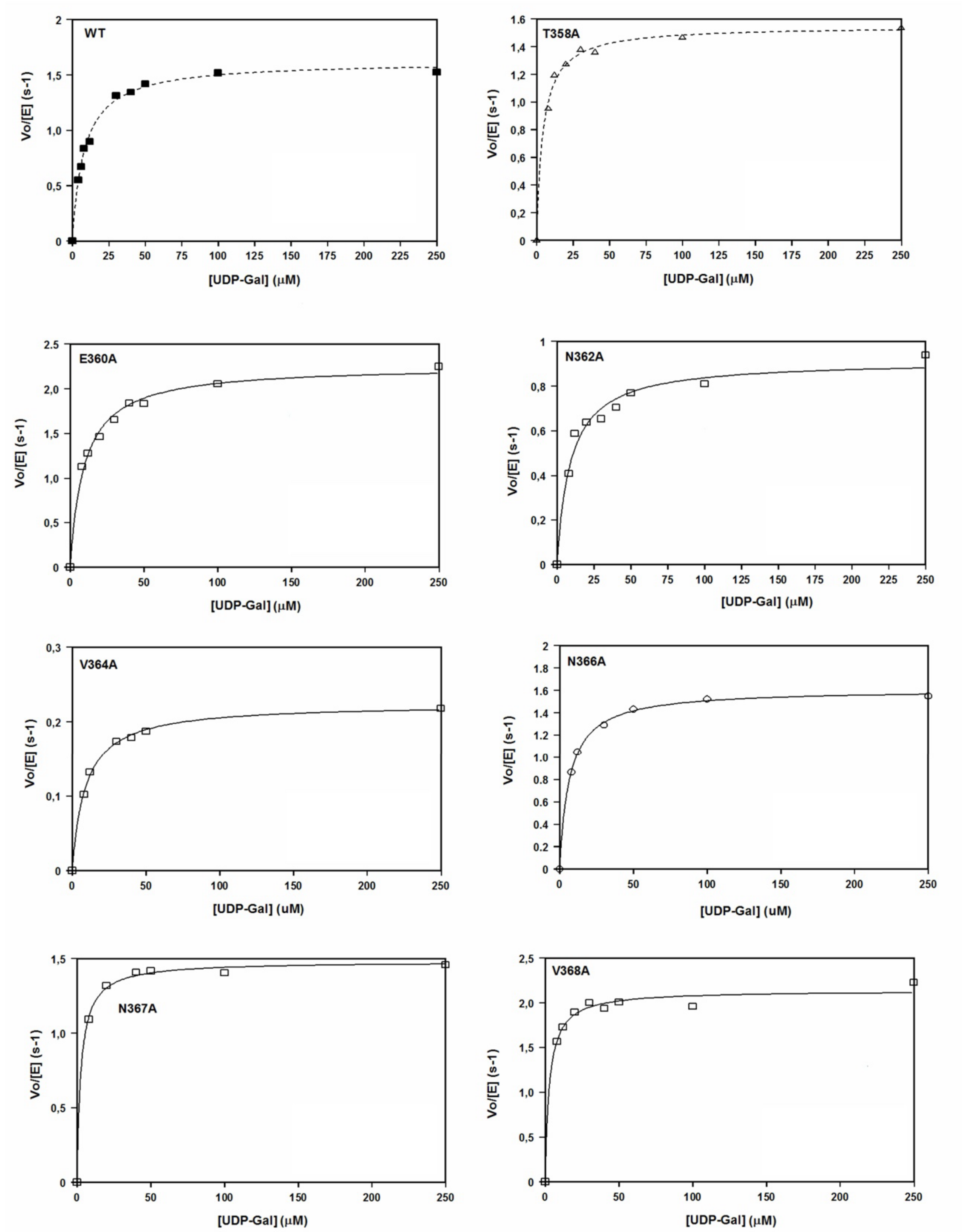

Figure S3. Kinetics of αGalT with acceptor substrate lactose at saturating (250 μM) donor (UDP-Gal) concentration.

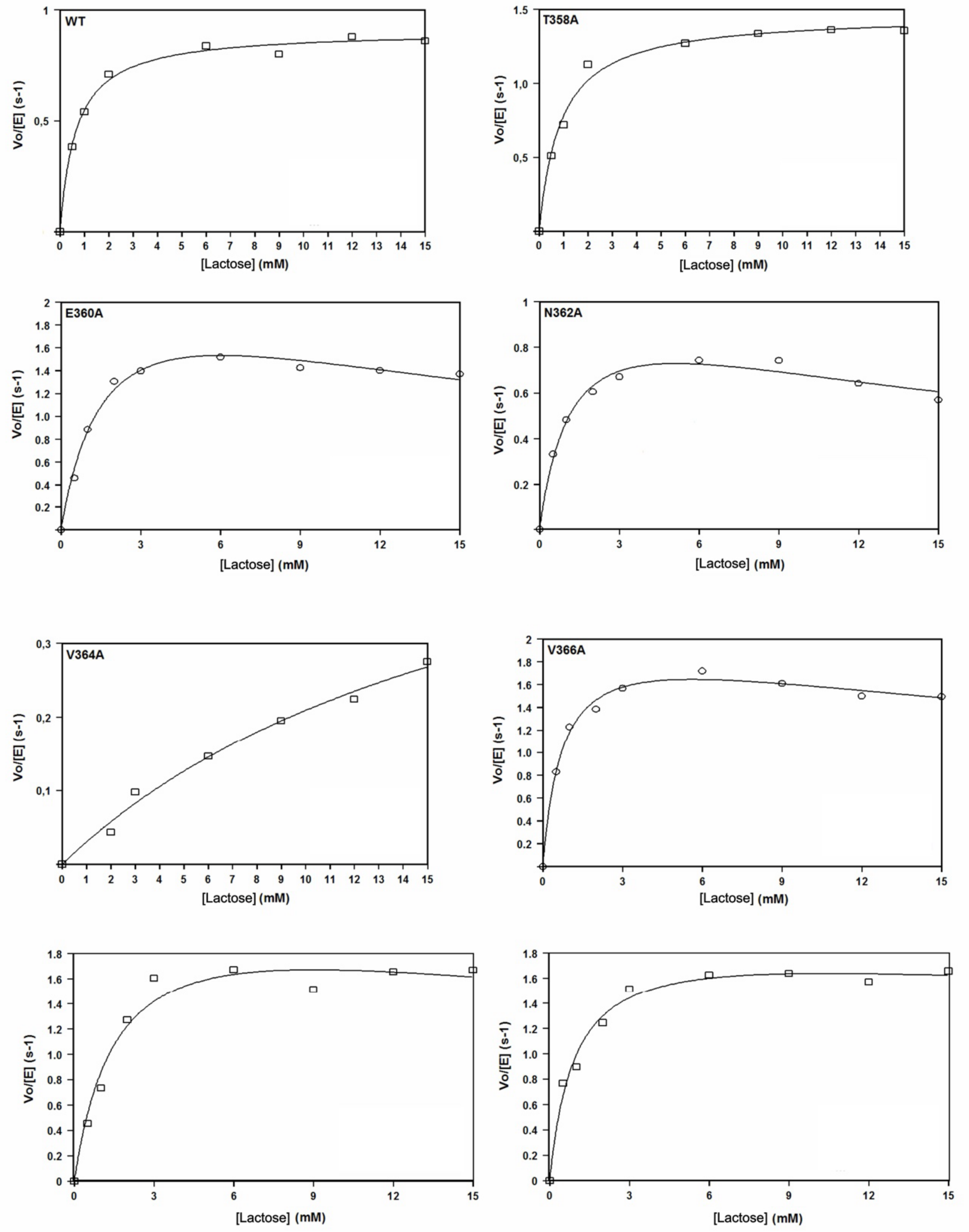

Figure S4. Urea-induced unfolding of αGalT wt and mutants in the absence and presence of UDP ligand (and UDP-Gal in the wt).

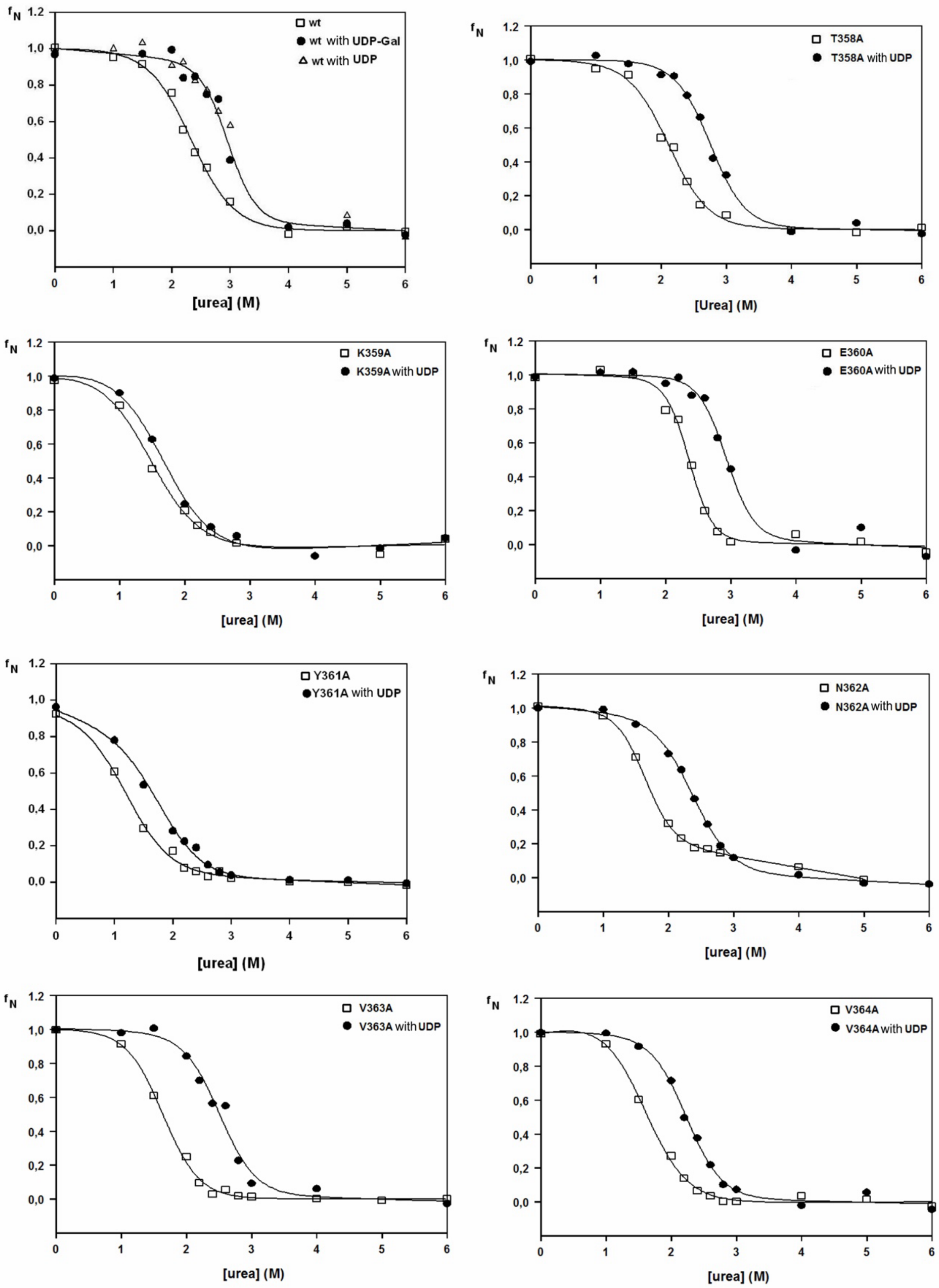

Figure S4-cont. Urea-induced unfolding of αGalT wt and mutants in the absence and presence of UDP ligand.

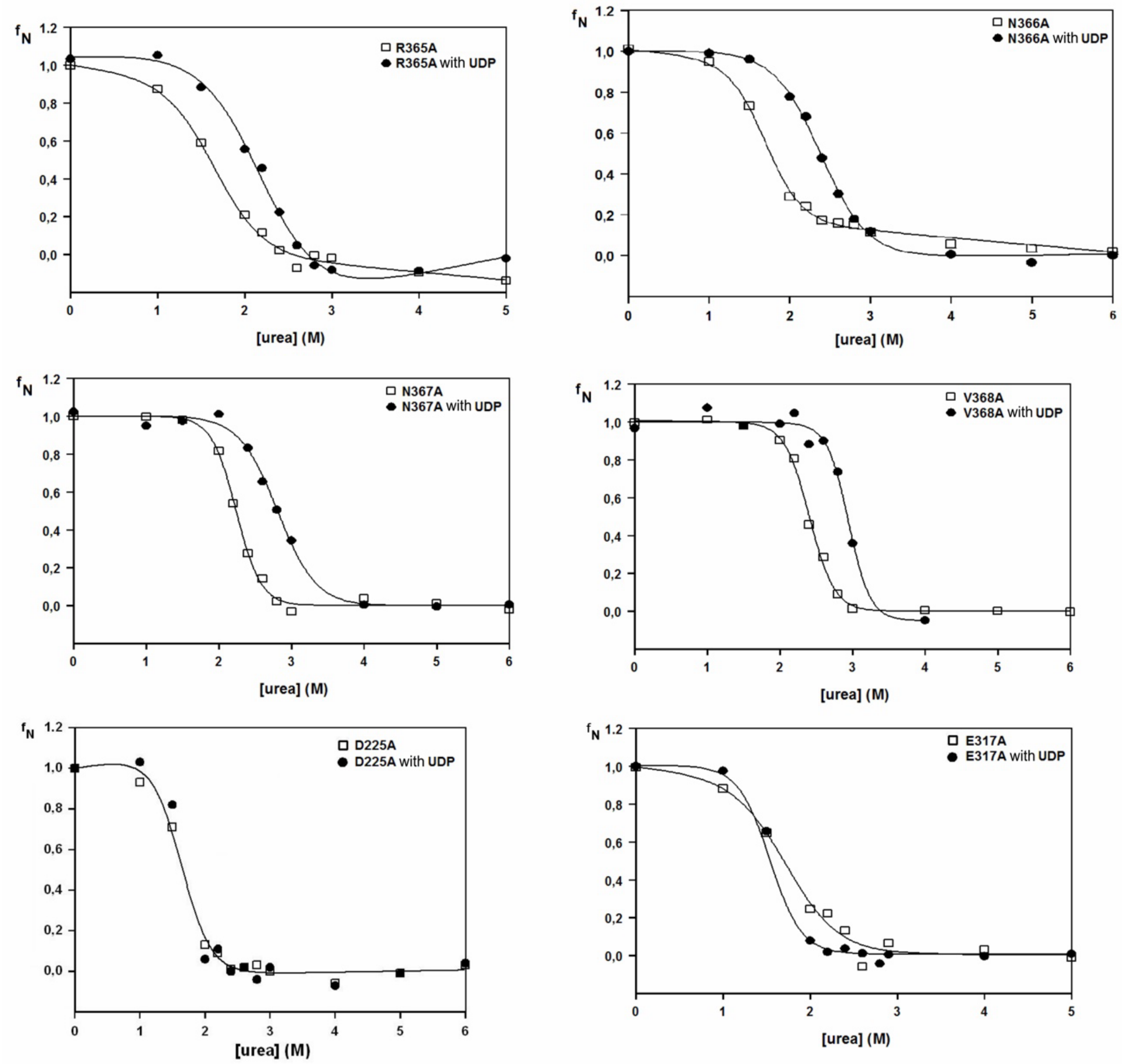

Figure S5. Conformational sampling of lactose and UDP-Gal during MD simulations of the α3GalT ternary complex. Superimposed representative ligand conformations extracted from the 200 ns MD simulations. The UDP-Gal nucleotide moiety oscillates between conformations resembling the activated Michaelis-complex-like 5NRD state (PDB: 5NDR) and the 5NRB-like state (PDB:5NBR). Carbon atoms of the 5NRD-like conformation are shown in blue, whereas carbon atoms of the 5NRB-like conformation are shown in brown.

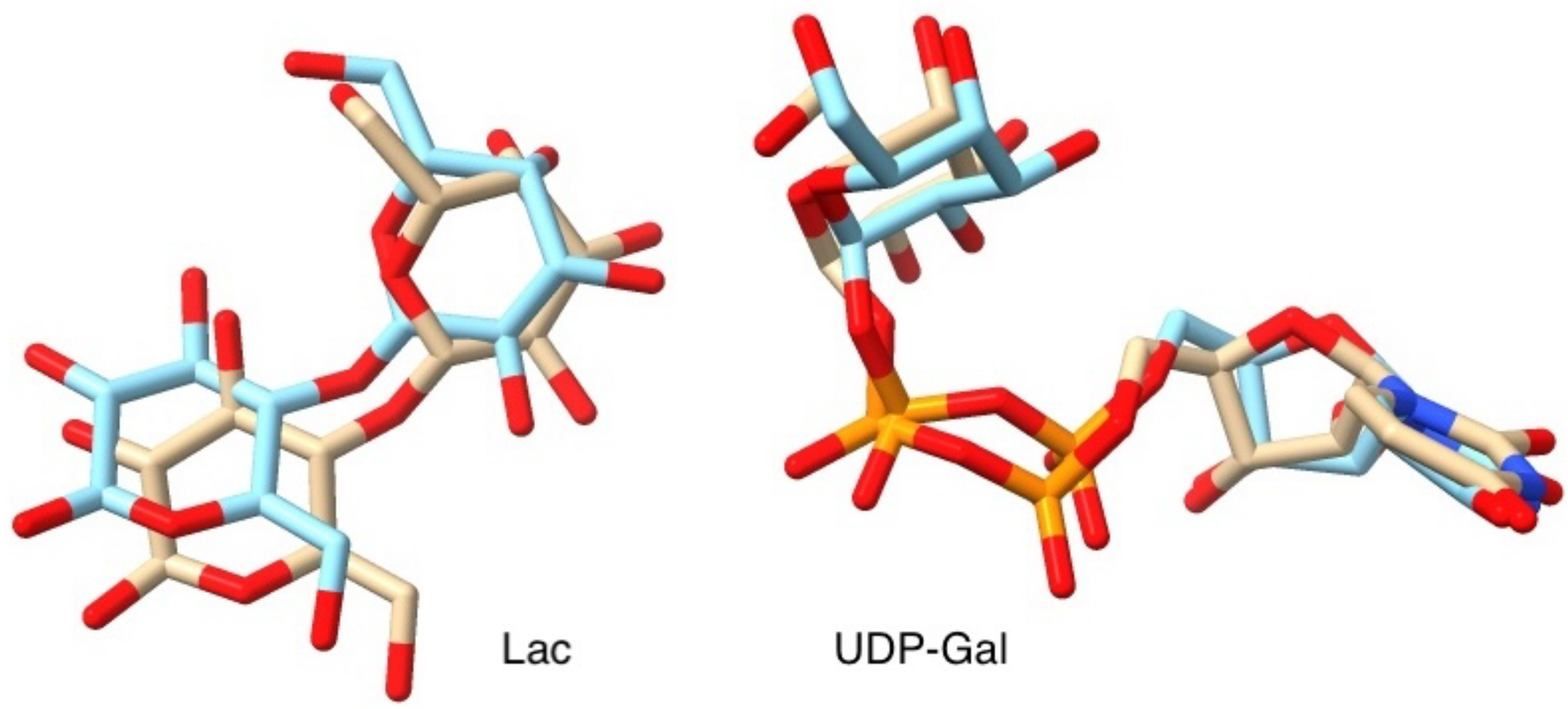

Figure S6. Inter-residue interactions in the closed conformation of the C-terminal helix in the Michaelis complex.

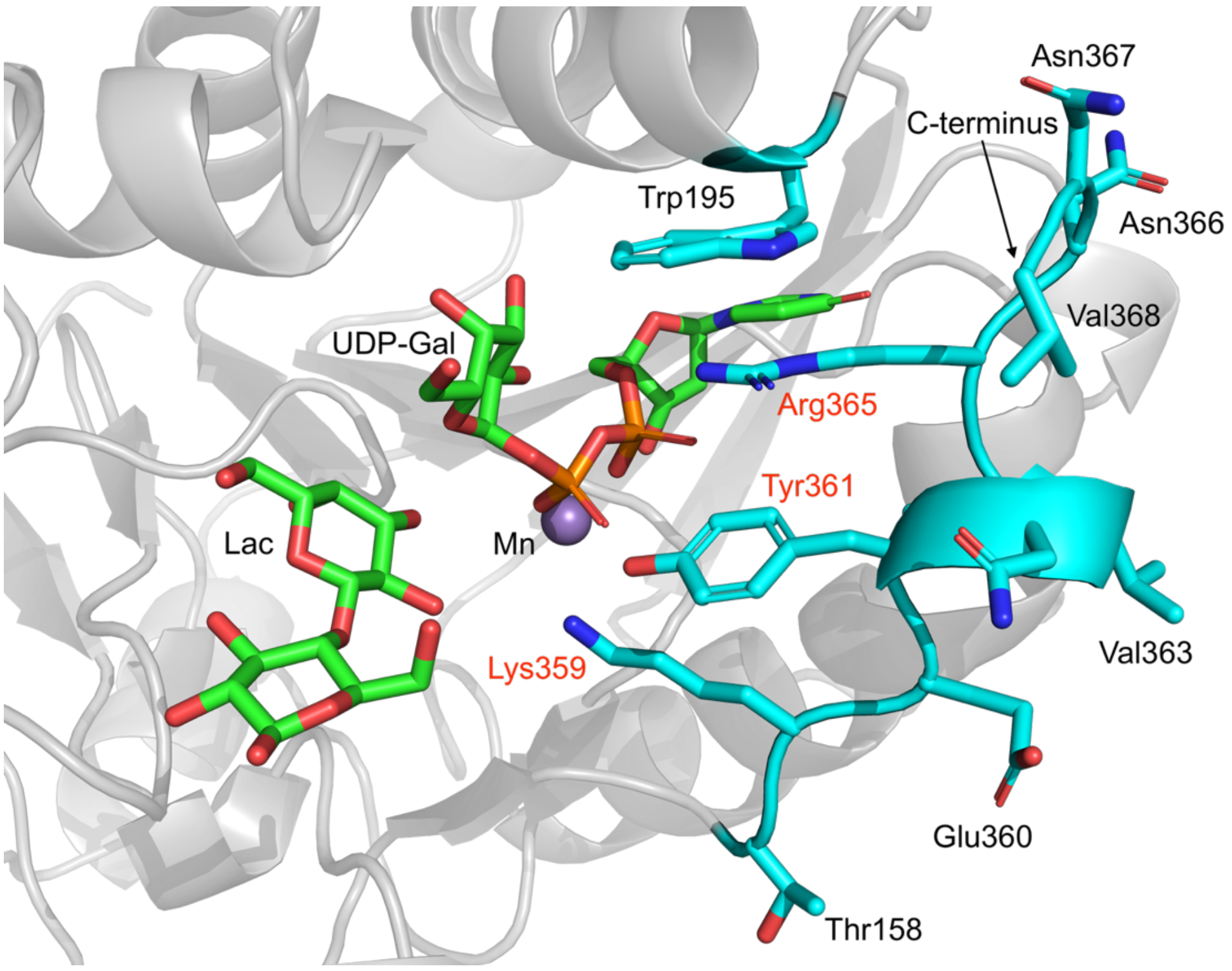

Supplement: Supplementary file 1 [file PRO-35-e70770-s001.pdf]
